# Real-Time Assistive System Integrating Geometric Topology Analysis and State-Adaptive Warning Logic for the Visually Impaired

**DOI:** 10.3390/s26123905

**Published:** 2026-06-19

**Authors:** Bilie Hu, Peishen Gao, Yan Liu, Xi Xia, Guoping Huo

**Affiliations:** School of Artificial Intelligence, China University of Mining and Technology-Beijing, Beijing 100083, China; 2310402111@student.cumtb.edu.cn (B.H.); 2310402103@student.cumtb.edu.cn (P.G.); 2310402116@student.cumtb.edu.cn (Y.L.); 2310402221@student.cumtb.edu.cn (X.X.)

**Keywords:** visually impaired assistance, assistive warning system, YOLOv9, tactile paving detection, geometric topology reconstruction, 1D connectivity clustering, finite state machine, embedded vision

## Abstract

Traditional white canes offer a limited perception range, whereas end-to-end visual models face challenges in real-time deployment on edge devices. To address these limitations, this paper proposes a lightweight real-time assistive system that integrates geometric topology reconstruction with state-adaptive warning logic. The system utilizes YOLOv9 to extract discrete semantic primitives of tactile paving. It constructs a dual-branch perception framework based on Median Absolute Deviation and the Minimum Spanning Tree algorithm to analyze the topological structure of tactile paving. For complex intersections characterized by warning indicators, a one-dimensional connectivity clustering algorithm based on longitudinal topology is proposed. It generates accurate macroscopic feasible directional prompts under field-of-view boundary constraints. Additionally, a hierarchical scheduling framework dynamically orchestrates scenario-specific finite state machines to enable continuous dynamic interaction across typical high-risk scenarios. Evaluated on a custom real-world dataset, the system achieves a 95.21% frame-level comprehensive accuracy for straight-path deviation correction and intersection directional prompting. Dynamic temporal stress tests confirm the temporal stability and logical coherence of state transitions. Furthermore, latency evaluations demonstrate the logic layer’s minimal computational overhead, proving its theoretical feasibility for real-time edge deployment. This approach provides an effective, low-latency solution for delivering directional prompts and hazard warnings to visually impaired users.

## 1. Introduction

Visual impairment has become a severe global public health challenge. Approximately 1.1 billion people globally faced vision loss by 2020, with the number of completely blind individuals reaching 43 million; due to an aging population, this figure is projected to exceed 60 million by 2050 [1]. Such impairments significantly limit the ability of affected individuals to live independently. Statistics indicate that nearly half of visually impaired individuals have never attempted independent travel due to safety concerns [2]. Traditional physical assistive tools, such as white canes, have a limited perception range and lack foresight, while guide dogs are constrained by high training costs. Therefore, developing Electronic Travel Aids (ETAs) equipped with environmental semantic understanding capabilities holds significant practical importance.

Despite the promising advancements in ETAs, there remains an inherent tension between lightweight model design for edge deployment and the rich semantic perception required for effective directional prompting. Visually impaired users depend on real-time, on-device solutions to avoid latency and reliance on network connectivity. At the same time, complex urban environments necessitate robust semantic perception to ensure safe and precise directional prompting. While existing approaches have achieved notable progress in specific sub-tasks, they generally lack a comprehensive, decoupled framework tailored for the multifaceted daily assistance of visually impaired individuals. Most current systems fail to simultaneously support lightweight edge deployment, robust semantic perception, and multi-hazard early warning within a cohesive pipeline.

To address these limitations, this paper proposes a real-time assistive system integrating geometric topology analysis and state-adaptive warning logic. Unlike existing works that rely purely on the computational power of heavy end-to-end visual models, this study constructs a hierarchical processing architecture comprising lightweight perception, geometric topology reasoning, and state-adaptive warning. The system employs lightweight YOLOv9 as the foundational perception module. Upon this foundation, a dual-branch algorithm is utilized to reconstruct the geometric topology of tactile paving. For complex intersections, a one-dimensional connectivity clustering algorithm based on longitudinal topology is proposed to generate real-time macroscopic feasible directional prompts under field-of-view boundary constraints. Furthermore, it incorporates scenario-specific finite state machines (FSMs) to establish an integrated temporal interaction system. This system aims to minimize computational complexity without compromising the accuracy of fine-grained scene understanding, effectively addressing the challenges of delivering safety prompts in complex road conditions and providing solid computational efficiency and theoretical feasibility for low-latency deployment on edge devices.

The main work and contributions of this paper are as follows:

- We construct a hybrid fine-grained dataset containing 24 categories of objects including tactile paving, obstacles, traffic signals, and stairs. For the core tactile paving task, a fine-grained annotation strategy at the instance level is implemented for individual tactile tiles, providing a high-quality discrete feature benchmark for subsequent geometric topology reconstruction. Meanwhile, diverse open-source data are integrated to provide reliable perceptual primitives for the subsequent multi-scenario hierarchical temporal interaction system.
- We propose a dual-branch geometric topology reconstruction algorithm for visual detection results. In straight-path scenarios, a robust line-fitting algorithm based on Median Absolute Deviation (MAD) eliminates outlier noise. In complex intersection scenarios, the Minimum Spanning Tree (MST) algorithm is introduced to reconstruct the topology skeleton, achieving mathematical analysis of structures such as branches and turns.
- To transform topology features into directional prompts, we design a scene-adaptive, dual-branch prompting mechanism based on geometric constraints. For paths consisting solely of directional indicators, we develop a dual deviation correction logic based on spatial constraints, prioritizing heading correction over lateral adjustment. For complex intersections characterized by warning indicators, we propose a one-dimensional connectivity clustering algorithm based on longitudinal topology. By utilizing Region of Interest (ROI) scanning, this algorithm generates accurate macroscopic feasible directional prompts, effectively resolving semantic analysis difficulties at multi-branch intersections.
- To enable continuous dynamic interaction, we develop a hierarchical scheduling framework that dynamically orchestrates multiple scenario-specific FSMs. By enforcing temporally consistent state transitions through noise filtering and state stabilization mechanisms, the proposed FSMs effectively overcome the memoryless limitation of single-frame visual detection. This design supports robust multi-scenario interaction in high-risk environments, including obstacle avoidance, stair negotiation, and street crossing, elevating the system from static topology reasoning to spatiotemporal dynamic interaction under edge deployment constraints.

The remainder of this paper is organized as follows. Section 2 reviews the related work in ETAs and highlights the specific research gaps addressed by the proposed approach. Section 3 details the proposed hierarchical assistive system, encompassing the theoretical semantic definitions, the dual-branch geometric topology reconstruction, and the state-adaptive warning logic driven by multi-scenario FSMs. Section 4 outlines the experimental methodology, including dataset construction, hardware setup, and evaluation protocols. Section 5 presents a comprehensive evaluation of the system, covering perceptual performance, geometric warning accuracy, dynamic temporal robustness, and computational efficiency. Section 6 discusses the overall effectiveness of the system, validates its feasibility for edge deployment, and identifies current limitations alongside future research directions. Finally, Section 7 concludes the paper.

## 2. Related Work

ETAs have evolved rapidly to enhance the spatial awareness and mobility independence of visually impaired people. Existing visual-based assistance frameworks generally fall into two primary technical paradigms: end-to-end heavy deep learning frameworks and pixel-level semantic segmentation methods. This section reviews these two streams and clarifies the distinct research gap that the proposed system aims to address.

### 2.1. End-to-End Deep Learning and Large Multimodal Models

Driven by recent breakthroughs in artificial intelligence, various end-to-end deep learning networks and vision-language models have been introduced into the ETA domain to deliver comprehensive environmental descriptions. These approaches transform first-person visual streams directly into natural language descriptions or macro-level navigation instructions [3,4]. For instance, studies have explored deploying vision-language models (VLMs) on smartphones to generate context-aware descriptions without relying on cloud infrastructure [3], while frameworks like NavGraph have been developed to parsimoniously orchestrate guidance instructions to prevent cognitive overload [4].

Although these systems demonstrate strong semantic understanding capabilities, their architectural complexity poses challenges for practical edge deployment. The large parameter scale and computational demands often lead to increased inference latency and energy consumption, making real-time execution difficult on resource-constrained wearable processors. As a result, balancing semantic perception capability with lightweight edge-side responsiveness remains an open challenge for assistive navigation systems.

### 2.2. Pixel-Level Semantic Perception for Tactile Paving

To achieve a better balance between latency and accuracy, another technical stream focuses on localized semantic perception, specifically target detection and semantic segmentation of tactile paving [5,6,7]. These methods utilize lightweight neural networks to classify and isolate tactile paving areas from standard sidewalk surfaces. For example, dual-branch architectures like DSC-Net [5] and other lightweight networks optimized for embedded sensors [6] have been proposed to extract global context and edge features of tactile paving in real-time. Similarly, optimized YOLO architectures have been utilized for infrastructure inspection tasks, balancing computational cost with detection precision [7].

While these models achieve strong performance in pixel-level and box-level perception tasks, they generally formulate scene understanding as an independent perception process without further structural reasoning. The generated bounding boxes or segmentation masks mainly describe local tactile paving regions, but provide limited support for constructing global topological relationships, estimating trajectory deviation trends, or generating high-level directional assistance. Moreover, under real-world conditions involving tactile tile degradation, shadow occlusion, and complex intersection layouts, perception outputs may become fragmented or spatially inconsistent. Without additional geometric refinement and temporal consistency modeling, maintaining stable and reliable assistive guidance in dynamically changing urban environments remains challenging.

### 2.3. Summary and Research Gap

In summary, existing ETA approaches generally exhibit a trade-off between semantic understanding capability and deployment efficiency on edge devices. Large end-to-end models provide richer scene interpretation but often impose substantial computational and latency overhead, whereas lightweight perception-based methods achieve efficient execution yet provide limited support for structured reasoning and continuous assistive prompting.

To address these limitations, this work proposes a hierarchical and decoupled assistive system. Instead of relying solely on computationally intensive semantic models or isolated pixel-level predictions, the proposed system strategically decouples deep learning-based visual perception from CPU-friendly geometric topology reasoning. By combining a lightweight instance-level tactile tile detector with robust double-branch geometric topology reconstruction and state-adaptive FSMs, the system achieves low-latency, real-time directional prompting and hazard warning without compromising safety or environmental semantic robustness under edge-computing constraints.

## 3. Materials and Methods

### 3.1. Theoretical Foundations and Semantic Definitions

To achieve the leap from visual perception to the topological understanding of complex path structures, the system must establish a rigorous, fine-grained semantic baseline. Conventional visual detection treats tactile paving as continuous regions. This representation often leads to feature merging at complex multi-branch intersections, thereby hindering the accurate parsing of topological directions. The research focuses on tactile paving as the core infrastructure for visually impaired individuals. It abandons holistic feature extraction and decomposes continuous road semantics into discrete instances of individual tiles. According to the national standard Codes for Accessibility Design (GB 50763-2012) [8], tactile ground surface indicators (TGSIs) are paving tiles designed to provide tactile feedback and guide pedestrians toward their destinations. The standard strictly categorizes these indicators into the following two semantic entities:

1. Directional Indicator: It features a striped surface and provides tactile feedback through a cane or footsteps, directing visually impaired pedestrians to walk straight ahead.
2. Warning Indicator: It features a dotted surface and serves to mark starting points, turns, endpoints, and facilities, or to alert pedestrians of potential hazards ahead.

**Figure 1:**
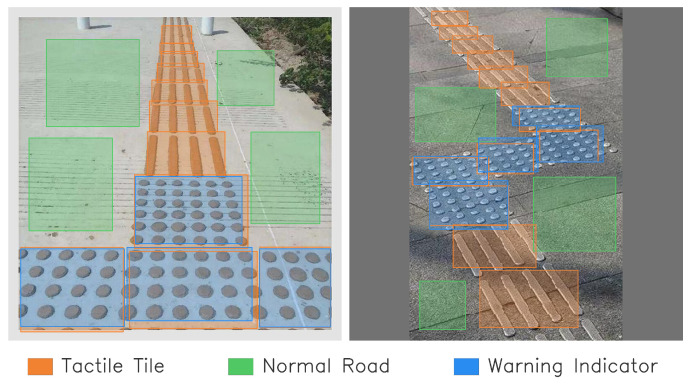
An example of instance-level tactile paving annotations.

Guided by these definitions, the annotation process classifies targets into general tactile tiles and specific warning indicators. It employs a fine-grained annotation strategy at the tile instance level, as illustrated in Figure 1. This discrete annotation strategy preserves the spatial arrangement and topological structures of the tiles. It provides the essential data foundation for subsequent topology reconstruction and path reasoning based on geometric constraints.

### 3.2. Geometric Topology Reconstruction and Warning Logic

To address the challenges of tactile paving semantic parsing and path deviation warning in assistive systems for the visually impaired, this paper proposes a hierarchical perception and warning system integrating geometric constraints and topological reasoning. The system first utilizes a lightweight network to extract initial semantic primitives and accurately decouples the directional indicator features based on spatial exclusion logic. Based on this foundation, it constructs a dual-branch parsing algorithm guided by the spatial topological priors of standard tactile paving [8]. For linear path segments consisting solely of directional indicators, the system quantifies pose deviations based on robust line fitting. For path segments that contain warning indicators and exhibit complex connectivity, the system employs an MST algorithm to reconstruct the topological skeleton. Furthermore, building upon these geometric and topological foundations, the system constructs a scene-adaptive warning algorithm. This algorithm integrates pose correction and topological semantics into guidance logic, providing a reliable mathematical baseline for safe multi-scenario interactions while preserving user decision-making autonomy.

#### 3.2.1. Prerequisite Assumptions

The geometric reasoning of the system relies on imaging and spatial priors. Fundamentally, the system operates under the assumption that the user starts within or adjacent to a tactile paving network, ensuring the continuous availability of valid semantic features to activate the visual processing pipeline. At the perception end, the camera height is set to approximately 1.6 m to capture a first-person visual perspective. It maintains a fixed downward pitch angle to form a continuous field of view covering the blind spot underfoot to the road ahead. Combined with a 1:1 distortion-free square field of view, this setup establishes a spatially explicit scale reference for monocular reasoning. At the algorithm end, the system introduces a topological linear prior for scenarios without warning indicators to support low-complexity fitting. Simultaneously, it constructs a normalized egocentric coordinate system with the origin at the bottom-left corner of the image. This coordinate system enables position parameters and slope signs to directly correspond to the directional tendencies in the physical space. Moreover, based on perspective projection relationships, it models the user’s location as the midpoint of the image’s bottom edge, establishing a standardized reference baseline for subsequent scene-adaptive geometric deviation calculations.

#### 3.2.2. Image Preprocessing

To ensure the consistency of subsequent geometric calculations, input video frames are standardized to a 640×640 resolution through distortion-free bi-linear interpolation. For the general tactile tile and warning indicator bounding boxes output by the lightweight network, the system performs geometric abstraction and semantic decoupling. It abstracts the general tactile tile bounding boxes into a set of geometric center points, and applies a spatial exclusion logic to strictly filter out any center points enclosed by warning indicator bounding boxes. This exclusion strategy effectively eliminates the semantic redundancy arising from the spatial overlap of both indicator types. It achieves a differentiated feature representation for directional indicators, modeled as a discrete set of keypoints, and warning indicators, modeled as warning boxes. This directly lays a structured geometric foundation for subsequent topological connectivity reconstruction and path reasoning.

#### 3.2.3. Topological Structure Analysis of Tactile Paving

An adaptive topology analysis strategy based on geometric configurations is proposed to map discrete semantic primitives into continuous walkable tactile paving. Given the significant structural variations of tactile paving in real-world scenarios, a single fitting algorithm struggles to balance computational robustness and accuracy. Therefore, the system utilizes the presence of warning indicators as a scenario classification prior to establish a dynamic branching mechanism. When only directional indicators are present within the visual field, indicating a linear extension of the tactile paving, the system applies a linear fitting algorithm. Conversely, when warning indicators are detected, the system infers non-linear connectivity ahead and employs a graph-theoretic path generation algorithm. This dual-branch strategy effectively synthesizes discrete geometric primitives into macroscopic path orientations.

(1) Robust Line Fitting for Linear Tactile Topologies

In scenarios where no warning indicators are detected, the system assumes the directional indicators exhibit a linear topology. A robust fitting algorithm combining principal direction determination and MAD [9] is proposed to efficiently extract the directional trend from the discrete keypoints. Compared to the classic RANSAC [10] algorithm relying on non-deterministic random sampling, this method achieves statistical filtering with a deterministic O(N) time complexity. It satisfies strict real-time requirements on edge devices while effectively eliminating the interference of perspective distortion and detection noise on the overall trend.

The algorithm first compares the spatial span of the feature point set along both coordinate axes to prevent numerical instability arising from the infinite slopes of vertical tactile paving. The axis exhibiting the larger span is designated as the independent variable for an initial least squares fitting, yielding a set of Euclidean distance residuals *R* from each point to the initial regression line. To account for scale variations induced by perspective effects, the MAD statistic is introduced to formulate an adaptive outlier rejection threshold *T*:

(1) T=Median(R)+λ·MAD

where MAD=Median(|R−Median(R)|) and λ is a hyperparameter modulating sensitivity. Based on extensive empirical testing, λ is optimized to 2.0, as this value optimally balances the preservation of valid tactile features with the effective filtration of spatial outlier noise. Upon filtering outliers with residuals exceeding this dynamic threshold *T*, a principal direction-based least squares fitting is re-applied to the retained inliers. Finally, the derived line equation is standardized with the *x*-axis as the independent variable (y=kx+b), yielding a highly robust directional equation for the tactile paving.

(2) Graph-Theoretic Topological Reconstruction for Complex Intersections

The appearance of warning indicators typically signifies the presence of non-linear structures such as bifurcations or curves ahead, rendering conventional linear regression ineffective. Therefore, a data-driven tactile paving skeleton reconstruction algorithm based on an MST is proposed, detailed in Algorithm 1. The algorithm first extracts the center points of all tactile paving detection boxes to construct a vertex set *V*, generates a weighted complete graph based on the Euclidean distance between points, and utilizes Kruskal algorithm [11] to adaptively reconstruct the topological skeleton. Addressing abnormal long edges induced by false detections in distant regions of the immediate visual field, the MAD statistic [9] is introduced to implement dynamic pruning. The median of MST edge lengths *M* is calculated, and an experimentally validated empirical constant of 2.5 is set to remove abnormal connections satisfying the following condition:

(2) $$l_{e}>M+2.5\times \mathrm{MAD}$$

This pruning algorithm effectively filters out outlier noise points, preserving a highly reliable topological backbone without the premise of predefined templates.

After extracting the clean topological skeleton, the system performs semantic classification of topological key points based on the vertex degree d(v) of the graph. Nodes with degrees of 1 and 2 represent endpoints and intermediate path points respectively, while the set of nodes satisfying d(v)≥3 is accurately identified as bifurcation points, such as the center of a T-junction or crossroad. Finally, using the bifurcation points as starting nodes, the system employs a depth-first search (DFS) strategy [12] to traverse the graph structure, decomposing the complex mesh topology into several independent walkable tactile paths. This reconstruction process provides precise spatial priors for the subsequent generation of feasible directional prompts at complex intersections, empowering visually impaired users to make autonomous directional decisions.
**Algorithm 1** Topology-aware tactile paving reconstruction**Require:** Tactile paving bounding boxes B**Ensure:** Topology type *S*, extracted walkable paths L   1:V←{center(b)∣b∈B}   2:$E_{\mathrm{MST}}\leftarrow \mathrm{Kruskal}\left(V,{∥u-v∥}_{2}\right)$ {MST Construction}   3:$M\leftarrow \mathrm{median}(\left\{l_{e}|e\in E_{\mathrm{MST}}\right\})$, $\sigma \leftarrow \mathrm{MAD}(E_{\mathrm{MST}})$   4:$E^{\prime }\leftarrow \left\{e\in E_{\mathrm{MST}}|l_{e}\leq M+2.5\times \sigma \right\}$ {MAD-Based Edge Pruning}   5:$P_{\mathrm{bifurcation}}\leftarrow \left\{v\in V|\mathrm{degree}\left(v,E^{\prime }\right)\geq 3\right\}$ {Bifurcation Node Identification}   6:$S\leftarrow \mathrm{Classify}\text{\_}\mathrm{Topology}(P_{\mathrm{bifurcation}},\mathrm{degree})$ {Linear, T-Shape, Cross}   7:$L\leftarrow \mathrm{DFS}\text{\_}\mathrm{Extract}\text{\_}\mathrm{Paths}(V,E^{\prime },P_{\mathrm{bifurcation}})$ {Walkable Path Extraction via DFS}   8:**return** S,L

#### 3.2.4. Tactile Paving Deviation Warning Algorithm

**Figure 2:**
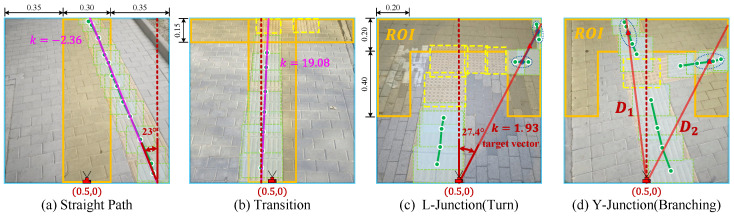
Visualization of geometric constraints and semantic perception logic. In the subfigures, the red dashed line represents the forward axis of the camera (user), while the red solid lines indicate the target vectors for directional prompts. The purple solid lines denote the robust fitted lines of the linear tactile topologies. Furthermore, the orange boxes highlight the Regions of Interest (ROIs), while the green points represent the extracted center points of the directional indicators, and the yellow dashed boxes indicate the warning indicators.

Building upon the acquired topological semantics of the tactile paving, a deviation warning algorithm based on geometric constraint perception is developed. This algorithm aims to establish a logical mapping mechanism from pure geometric topology analysis to spatial warning directives. The scenario-adaptive geometric perception logic is illustrated in Figure 2. Given the significant structural variations of tactile paving in real-world scenarios, the guidance logic employs a context-adaptive processing scheme. In straight-ahead scenarios without warning indicators, the system focuses on real-time pose correction. It mitigates deviation risks by quantifying heading angle errors and lateral offsets. In complex intersection scenarios containing warning indicators, the focus shifts to macroscopic directional prompts driven by a 1D connectivity clustering algorithm under field-of-view boundary constraints. By analyzing the extension trend of the tactile paving in the distant field of view, the system extracts and provides real-time feedback on all feasible branch directions. This ensures physical interaction safety while maximizing the independent decision-making capability of visually impaired users.

(1) Linear Tactile Paving: Dual-Constraint Deviation Correction

In standard walking scenarios containing exclusively directional indicators, the tactile paving exhibits linear geometric features under perspective projection. The system derives critical geometric warning thresholds based on a walking reaction model. Standard directional indicators typically consist of two 0.25 m × 0.25 m tactile tiles laid side-by-side representing a total width of 0.5 m [8]. Thus, a rigorous half-width safety margin $M_{\mathrm{safe}}=0.25$ m is established for the tactile paving. Considering a user reaction adjustment distance $D_{\mathrm{react}}\approx 1.5$ m which corresponds to approximately two normal steps, the geometric relationship $\tan\left(\theta_{\max}\right)=M_{\mathrm{safe}}/D_{\mathrm{react}}\approx 0.167$ yields a maximum allowable yaw angle $\theta_{\max}\approx 9.46^{\circ }$. This corresponds to a critical slope value $T_{k}=6$ in the normalized coordinate system. Furthermore, based on perspective projection mapping with a camera height H=1.6 m and a horizontal field of view $\mathrm{FOV}\approx 80^{\circ }$, the physical coverage width at the image bottom is calculated to be approximately 2.7 m, denoted as $2\cdot H\cdot \tan(40^{\circ })\approx 2.68$ m. To accommodate torso sway during walking, a tolerance buffer is introduced, expanding the standard normalized width of the tactile paving from 0.5/2.68≈0.19 to 0.3. This defines a high-precision safety interval of [0.35,0.65] centered at the image axis x=0.5. The corresponding geometric mapping is shown in Figure 2a.

A hierarchical deviation correction algorithm prioritizing heading over lateral position is designed according to the aforementioned geometric constraints. The first-priority heading deviation warning evaluates the line slope *k*. If |k|<6, as illustrated by the deviation state k=−2.36 in Figure 2a, the heading angle exceeds the safety threshold. This triggers an immediate rotational adjustment prompt, where 0<k<6 indicates a right turn and −6<k<0 indicates a left turn. The second-priority lateral deviation warning activates only when the heading satisfies the safety constraint |k|≥6. The system calculates the horizontal projection interval $\left[x_{\min},x_{\max}\right]$ of the fitted line on the x-axis. A left or right translation prompt is triggered when $x_{\min}<0.35$ or $x_{\max}>0.65$, respectively, while a projection fully contained within [0.35,0.65] indicates no lateral deviation. This hierarchical logic possesses strict mathematical completeness: under the heading constraint |k|≥6, the maximum horizontal projection span of the fitted line within the image frame is bounded by $\Delta x_{\max}=1/\left|k\right|\approx 0.167$. This span is strictly smaller than the 0.3 width of the safety interval. This theoretically eliminates the risk of issuing contradictory lateral prompts simultaneously, thereby ensuring the robustness of the warning algorithm.

(2) Complex Intersections: Topology-Aware 1D Clustering for Directional Prompts

For complex intersection structures indicated by warning indicators, a macroscopic feasible directional prompt algorithm driven by topology-aware 1D clustering is proposed. This algorithm is grounded in a core geometric prior: under first-person perspective projection, effective feature points at the field-of-view boundaries can accurately indicate the physical extension of tactile paving. To suppress the cognitive load induced by distant noise, a conditionally triggered far-field filtering mechanism is introduced. A far-field depth threshold of 0.85 is established, corresponding to a physical distance of approximately 4 m. When all warning indicator center points in the field-of-view remain beyond the predefined far-field threshold while directional indicators are still observable in the near field, the system determines that the user has not yet reached the intersection, as shown in Figure 2b. At this stage, the system shields distant warning signals and reverts to the straight-ahead deviation correction logic.

To extract valid spatial inputs for the 1D clustering algorithm, the ROI partitioning mechanism activates only upon confirming arrival at the intersection. To overcome anchor box drift and branch adhesion caused by perspective distortion, the algorithm constructs three mutually exclusive ROI sub-areas within the normalized image space. Lateral turn zones are strictly confined to the mid-ground interval to filter out interference from the main tactile paving in the immediate foreground. The boundary span of each region is uniformly set to 0.2 to prevent semantic confusion. The specific spatial constraints are defined as follows:

- Left turn region ($R_{L}$): x∈(0,0.2),y∈(0.4,0.8);
- Forward straight region ($R_{F}$): x∈(0,1),y∈(0.8,1);
- Right turn region ($R_{R}$): x∈(0.8,1),y∈(0.4,0.8).

A strict category constraint mechanism is implemented to eliminate the risk of misleading guidance. The region $R_{F}$ responds to any category of tactile paving to maintain forward continuity judgments. Conversely, $R_{L}$ and $R_{R}$ only respond to directional indicator features. This ensures that only directional features signify genuine lateral extensions, whereas warning indicators merely prompt attention without indicating feasible paths, as illustrated by the ROI scanning logic in Figure 2c,d.

A one-dimensional connectivity clustering algorithm based on longitudinal topology is designed for detection boxes satisfying semantic constraints within the target regions, as detailed in Algorithm 2. Initially, the algorithm extracts the center points of these detection boxes and sorts them in ascending order of their vertical coordinates to reconstruct the physical extension topology of the tactile paving. It subsequently traverses the point sequence to compute adjacent distances $d_{i}$ and introduces a connectivity determination threshold δ=0.3. Under the perspective projection scale, this parameter reliably distinguishes distinct topological branches while remaining robust to anchor box center drift induced by a single curved tactile paving. A distance $d_{i}<\delta$ indicates topological connectivity, assigning the points to the same cluster. Otherwise, it is treated as a spatial break, instantiating a new cluster. The algorithm calculates the geometric mean of points within each cluster to serve as the directional target $P_{target}$ for that branch. Compared to methods that rely on predefined branch numbers, such as K-Means [13] and Agglomerative clustering [14], as well as density-based approaches lacking perspective priors, such as Mean Shift [15] and DBSCAN [16], the proposed method leverages longitudinal topological continuity to reduce a two-dimensional clustering problem to an O(NlogN) complexity, dominated by the initial coordinate sorting step, followed by a linear O(N) traversal. This strategy adaptively handles an unknown number of intersection branches, significantly reducing inference latency on edge devices, while maintaining robustness to non-uniform feature point density induced by monocular perspective effects.

After obtaining the target centroid $P_{\mathrm{target}}$, a guidance vector directed from the reference point $P_{\mathrm{user}}\left(0.5,0\right)$ to $P_{\mathrm{target}}$ is constructed, with slope *k*. The yaw angle θ is then computed as $\theta =90^{\circ }-\arctan\left(|k|\right)$. A negative slope (k<0) indicates a left turn of θ, whereas a positive slope (k>0) indicates a right turn of θ.

The system adopts a cross-region path fusion mechanism for scenarios with multiple activated regions. A fusion threshold ϵ=0.2, smaller than the intra-cluster threshold δ=0.3, is defined to prevent spurious topological merging of closely spaced bifurcations through stricter spatial constraints. Adjacent region centroids with spatial distances smaller than the fusion threshold are merged based on topological continuity, yielding a single continuous trajectory and a unified guidance vector, as illustrated in Figure 2c. In contrast, centroid distances larger than the fusion threshold indicate a multi-branch intersection, as shown by branches $D_{1}$ and $D_{2}$ in Figure 2d. In such cases, the system sequentially outputs all feasible directions. The system focuses on providing macroscopic feasible directional cues, leaving the final path decision to the user.
**Algorithm 2** Topology-aware 1D clustering and path guidance**Require:** Dets D, ROIs $\left\{R_{L},R_{F},R_{R}\right\}$, Thres δ=0.3,ϵ=0.2**Ensure:** Guidance vector set V   1:T←∅, $P_{\mathrm{user}}\leftarrow \left(0.5,0\right)$   2:**for all** region $r\in \{R_{L},R_{F},R_{R}\}$ **do**   3: S←{p∈D∣p∈r∧ValidClass(p,r)}   4: **if** S=∅ **then**   5:  **continue**   6: **end if**   7:$\;(p_{1},\ldots ,p_{n})\leftarrow \mathrm{SortByY}\left(S\right)$   8:$\;C\leftarrow \{p_{1}\}$, C←∅ {1D Connectivity Clustering}   9: **for** i=2 **to** *n* **do**  10:   **if** $∥p_{i}-p_{i-1}∥\;<\delta$ **then**  11:    $C\leftarrow C\cup \{p_{i}\}$ {Topologically connected}  12:   **else**  13:    C←C∪{C}; $C\leftarrow \{p_{i}\}$ {Spatial break}  14:   **end if**  15: **end for**  16: T←T∪{Mean(c)∣c∈C∪{C}}  17:**end for**  18:$T^{*}\leftarrow \mathrm{CrossRegionPathFusion}\left(T,\epsilon \right)${Merge regions with distances <ϵ}  19:$V\leftarrow \{\mathrm{ComputeVector}\left(P_{\mathrm{user}},P_{\mathrm{target}}\right)|P_{\mathrm{target}}\in T^{*}\}$  20:**return** V

### 3.3. Multi-Scene Temporal–Interactive System Design

**Figure 3:**
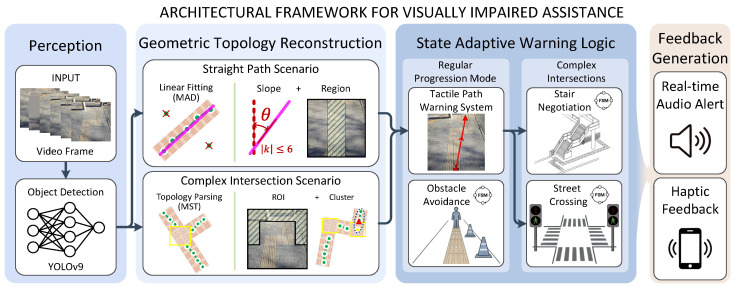
Overview of the system workflow.

The aforementioned geometric topology reconstruction and deviation warning algorithms establish the foundation for a state-adaptive warning system oriented towards dynamic temporal interaction. Its core logical flow is illustrated in Figure 3. To address the memoryless nature of single-frame detection, the proposed system incorporates multiple FSMs tailored to different scenarios. For high-risk scenarios such as obstacle avoidance, stair negotiation, and street crossing, the proposed system maps discrete visual detection primitives into a coherent sequence of discrete states under scenario-specific FSMs, incorporating noise-filtering and state stabilization mechanisms. This design enables a transition from static topological reasoning to dynamic autonomous interaction cycle.

#### 3.3.1. Decoupled Perception and Hierarchical Scheduling

Achieving robust semantic understanding of complex environments under edge computational constraints remains challenging. The system therefore adopts a decoupled dual-camera perception setup combined with a hierarchical state processing mechanism. The ground perception stream extracts tactile paving features and near-field semantic entities, while the environmental signal stream captures far-field traffic states. At the temporal interaction level, the system establishes a hierarchical processing mechanism encompassing perception, analysis, and dispatch. Accounting for both edge-computing constraints and processing latency, the global temporal resolution is standardized to T = 100 ms (i.e., 10 FPS). This establishes a rigorous time-domain baseline for state transitions and hysteresis-based state stabilization.

To ensure unambiguous guidance and user safety, the system enforces a mutually exclusive and strictly prioritized switching logic among the FSMs. During conventional progression in standard non-intersection zones characterized by continuous tactile paving, the primary thread executes geometric topology reconstruction and deviation warning, while a concurrent thread simultaneously executes the obstacle avoidance FSM. However, once a topological interruption is detected—indicating a potential intersection or structural breakpoint—the hierarchical scheduler forcibly suspends these routine states to prevent conflicting prompts. The system then executes a targeted hazard evaluation based on strict spatial urgency. It first prioritizes the extraction of stair features; if detected, the system immediately transitions to the stair negotiation FSM. If no stairs are identified, it activates the environmental signal stream to analyze traffic states, transitioning to the street crossing FSM if applicable. If neither of these specific high-risk semantics is detected, the system smoothly falls back to output a simple tactile paving interruption prompt, leaving the final navigation decision to the user. This layered scheduling mechanism guarantees low-latency, conflict-free state transitions in complex scenarios.

#### 3.3.2. Obstacle Avoidance FSM Based on Spatial Constraints

An independent obstacle avoidance FSM, invoked as a high-priority interrupt and executed in parallel with deviation warning while remaining mutually exclusive with other scenario-specific FSMs, handles occlusions caused by static objects and the instantaneous intrusion of dynamic entities during regular progression along tactile paving. This FSM is built upon an extended set of detection categories encompassing 15 typical obstacle classes.

Its internal state transition mechanism comprises four discrete states. The logical evaluation for these transitions is predicated on two core spatial constraint sets:

- Monitoring Region: $R_{\mathrm{mnt}}=\left[0.3,0.7\right]\times \left(0,0.7\right]$;
- Safe Critical Region: $R_{\mathrm{crit}}=\left[0.3,0.7\right]\times \left(0,0.3\right]$.

These regions define an effective passage width of approximately 1.0 m and a minimum braking safety margin. Within the initial idle state $S_{\mathrm{lock}}$, the bottom-center points of all detected obstacle bounding boxes are extracted as references. The FSM transitions to the planning state $S_{\mathrm{plan}}$ if and only if an obstacle enters $R_{\mathrm{crit}}$ or enters $R_{\mathrm{mnt}}$ and intersects the estimated tactile paving geometry. Upon transition, it locks onto the nearest obstacle $T_{m}$, defined as the target satisfying the aforementioned conditions with the minimum longitudinal coordinate.

**Phase 1. Spatial evaluation and feasible direction prompting:** This phase is implemented to prevent secondary collision risks in multi-target environments. The algorithm calculates the directional deflection angles, $\theta_{L}$ and $\theta_{R}$, from the forward vertical axis (x=0.5) to the bottom-left and bottom-right corners of the bounding box for the primary target $T_{m}$. By incorporating a tolerance angle $\delta =5^{\circ }$ to compensate for boundary jitter, the algorithm prioritizes constructing a virtual safe sector $R_{\mathrm{safe}}=\left[\theta_{\min}-\delta ,\theta_{\min}+\delta \right]$ along the side with the smaller deflection angle, designated as $\theta_{\min}$. An unobstructed path is confirmed if no secondary obstacles invade the monitoring region $R_{\mathrm{mnt}}$ within this virtual sector. The FSM then issues a directional avoidance prompt and transitions to the monitoring state $S_{\mathrm{monitor}}$. If the initial sector is obstructed, the FSM sequentially evaluates the alternative sector. Should both sectors prove impassable, it triggers a highest-priority emergency braking command.

**Phase 2. Continuous safety monitoring and smooth state reset:** During the avoidance execution phase, the algorithm dynamically refreshes the $R_{\mathrm{safe}}$ boundary based on the aforementioned spatial evaluation. When this sector aligns with the forward axis, the algorithm determines that the turn is complete, triggering the FSM to issue a straight-ahead passing prompt. Concurrently, a continuous, high-priority monitoring mechanism guarantees safety by triggering an immediate braking prompt whenever a new target breaches the critical region $R_{\mathrm{crit}}$. Once the algorithm determines the obstacle has been successfully bypassed, the state transitions to $S_{\mathrm{recover}}$. The FSM then issues a reverse rotation prompt (−θ) to correct the heading deviation induced by the avoidance maneuver. Simultaneously, a 20-frame hysteresis buffer (≈2.0 s) is activated. This temporal buffer provides the user with sufficient time to physically return to the tactile paving, facilitating a smooth reset to the conventional deviation warning mode.

#### 3.3.3. Stair Negotiation FSM with Temporal Hysteresis

The assistive system handles stair negotiation using a two-state FSM enabled by an extended stair-aware perception module. The trigger mechanism follows the standard environmental layout priors [8] by modeling a three-stage spatial adjacency relation, consisting of the interruption of directional indicators, the buffering of warning indicators, and the stair entities, which together serve as a spatial constraint. The FSM uses the interruption of the tactile paving coupled with warning indicators and the emergence of stair features as joint spatial conditions. This design acts as a noise filter and stabilizes state transitions, suppressing transient perception errors and ensuring a smooth transition into the stair negotiation mode.

Upon activation, the FSM suspends tactile paving detection and introduces a suppression window of 30 frames (≈3.0 s). This temporal mechanism strictly accommodates the gait transition time of visually impaired users, effectively shielding against premature state rollbacks caused by residual tactile paving features within the field of view before the user fully steps onto the stairs. Once the hysteresis concludes and re-evaluation confirms the user’s re-entry into the tactile paving, the system triggers a completion prompt and resets the FSM. Throughout this active state, the FSM internally executes a localized adaptive obstacle avoidance sub-routine. By applying the aforementioned spatial constraints, it issues decelerated-following or side-switching commands based on the semantic category of obstacles intruding into the monitoring region $R_{\mathrm{mnt}}$. The integration of this temporal control and concurrent logic completes the safety loop for stair scenarios with minimal system overhead.

#### 3.3.4. Street Crossing FSM Driven by Dual-Camera Synergy

To address complex intersections characterized by tactile paving interruptions, the system constructs a FSM to govern traffic light detection and temporal street-crossing guidance, leveraging dual-camera visual synergy. Building semantic priors through integrating open-source traffic elements including traffic lights and crosswalks within the perception module, this FSM encompasses idle monitoring ($S_{\mathrm{idle}}$), signal detection ($S_{\mathrm{detect}}$), crossing guidance ($S_{\mathrm{crossing}}$), and crossing completion ($S_{\mathrm{complete}}$). Given the inherent limitation of a ground-facing perspective in capturing distant signals, the FSM incorporates an environmental camera with a parallel field of view. A time-interleaved dual-camera scheduling strategy alternates between the ground perception stream and the environmental signal stream on a frame-by-frame basis. This mechanism enables concurrent perception of traffic lights and ground conditions without increasing the overall computational budget.

**Phase 1. Temporally Feasible Green Light Determination:**

During the $S_{\mathrm{detect}}$ phase, to mitigate misjudgments caused by signal flickering, the FSM temporarily suspends semantic reasoning of the ground perception stream and performs continuous tracking of the environmental signal stream. Under non-red states, a 20-frame (≈2.0s) observation window is initiated to ensure coverage of at least two complete blinking cycles at the end of the green phase. A feasible green light is confirmed and the FSM transitions to $S_{\mathrm{crossing}}$ if the green light active duration ratio exceeds 80% and the number of green-state transitions (i.e., on–off switching events) is no greater than two. Otherwise, a higher transition count is interpreted as end-of-phase green light blinking, which triggers a forced waiting period of 80 frames (≈8.0s), after which the green light evaluation process is restarted with a new observation window. After safe passage is confirmed, a minimum crossing timer is activated synchronously to prevent premature FSM rollbacks induced by residual proximal tactile paving features during the initial crossing phase. Under extreme conditions involving prolonged signal unidentifiability, a degradation mechanism triggers a fallback to the tactile paving deviation warning logic to ensure the system’s robustness.

**Phase 2. Crossing Assistance and State Recovery Mechanism:**

Upon entering the $S_{\mathrm{crossing}}$ state, the FSM performs lateral deviation correction using crosswalk detection results as spatial references, while continuously analyzing traffic light states to provide dynamic speed guidance. Concurrently, a crowd-aware pedestrian-following strategy is employed throughout the entire crossing process independently of traffic light states. Detecting pedestrians within the monitoring region $R_{\mathrm{mnt}}$ activates this following mechanism based on crowd behavior priors, maintaining a safe crossing status. The intrusion of an obstacle into the critical safety region $R_{\mathrm{crit}}$ strictly overrides this following behavior and triggers high-priority obstacle avoidance braking. This concurrent strategy establishes decision-level redundancy by leveraging crowd behavior priors, effectively compensating for transient signal loss and enhancing overall system robustness.

After the minimum crossing timer expires, tactile paving feature retrieval is reactivated to ensure a prompt state transition. The re-detection of tactile paving features triggers a transition to $S_{\mathrm{complete}}$, while crosswalk-based correction is maintained to prevent final-stage trajectory deviation. Once the user is confirmed to have entered the opposite tactile paving safe zone, the FSM completes state reset and smoothly transitions back to the regular tactile paving deviation warning mode.

## 4. Methodology

**Figure 4:**
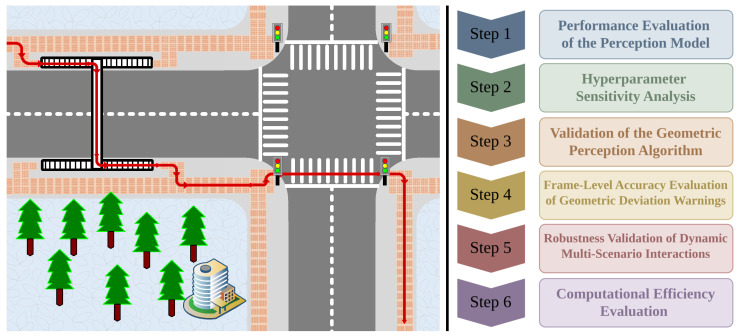
Simplified 2D urban street map illustrating the key testing scenarios, including straight paths, overpasses, and complex intersections (**left**), and the systematic six-step evaluation workflow (**right**). The red polyline further denotes the video data collection route used for system-level evaluation, covering representative urban mobility scenarios along the testing path.

To comprehensively evaluate the proposed hierarchical assistive system, a rigorous methodology is established. This section details the experimental setup, including dataset construction and hardware configurations, followed by the specific experimental procedures. As illustrated in Figure 4, the left panel presents a simplified 2D urban street map including key testing scenarios (e.g., straight paths, complex intersections, and overpasses) with red lines delineating the designated evaluation routes, while the right panel outlines a systematic six-step evaluation workflow, progressing from foundational perception to dynamic multi-scenario interactions and computational efficiency analysis.

### 4.1. Experimental Setup

To address the complexity and dynamic nature of real-world mobility environments for visually impaired individuals, a comprehensive dataset integrating various sidewalk elements and traffic scene information is constructed. This dataset comprises 24 object categories broadly divided into four modules: tactile paving, obstacles, traffic signals, and stairs. To jointly address scenario specificity and semantic generalization, a total of 7243 images containing tactile tiles are independently collected under diverse viewpoints and varying illumination conditions, and manually annotated at the instance level. These images are subsequently merged with high-quality publicly available annotated datasets and randomly split into training and validation subsets with an 8:2 ratio. An independent test set is further collected by members of the research team in real-world road environments, strictly adhering to a standardized camera installation configuration. During data preparation, all images undergo adaptive symmetric padding and are uniformly resized to a resolution of 640×640, ensuring consistent geometric space representation.

The experiment adopts a cloud-training and local-inference testing architecture. Model training is conducted on a server equipped with dual NVIDIA RTX 4090 GPUs. Inference and latency evaluations are performed on a mobile workstation equipped with an Intel Core i9-13980HX CPU and an NVIDIA RTX 4080 Laptop GPU. Although this testing platform provides substantial computational resources rather than being a resource-constrained edge device, this setup establishes a strict computational baseline. By isolating the latency of the CPU-bound logic layer on a known hardware configuration, the experimental design focuses on quantitatively demonstrating the extreme efficiency of the proposed lightweight geometric logic. The resulting baseline metrics substantiate the practicality of deploying the assistive system on actual edge platforms in the future.

The lightweight YOLOv9 [17] is adopted as the front-end perception model to achieve a balance between high frame rates and detection accuracy. As this study focuses on the engineering deployment of the assistive system rather than architectural redesign, the network structure is kept unchanged. Targeted parameter optimization is specifically designed to address both the strict requirements for high-precision detection and the long-tail distribution inherent in real-world mobility scenarios, where high-frequency categories such as tactile tiles and pedestrians vastly outnumber low-frequency classes including warning indicators and stairs. A class-balanced sampling strategy is employed to enhance feature extraction for underrepresented classes by increasing their sampling weights. Furthermore, a two-stage data augmentation scheme is implemented: mosaic augmentation is applied during the early training phase to improve background diversity and robustness, but is subsequently disabled in the final epochs to prevent artificial boundary artifacts from degrading bounding box regression accuracy.

### 4.2. Experimental Procedures

The experimental framework is designed to comprehensively evaluate the proposed system in terms of perceptual performance, algorithmic robustness, temporal stability, and computational efficiency. Accordingly, the evaluation is organized into the following six experimental protocols.

Performance Evaluation of the Perception Model: To establish a computational baseline for the overall system architecture, the trained front-end perception model is evaluated on the validation set containing all 24 categories. The quantitative evaluation metrics focus on precision (P), recall (R), and mean average precision at a 50% intersection over union threshold (mAP@0.5). Furthermore, a qualitative assessment is conducted on real-world test sequences to evaluate the model’s spatial localization robustness under challenging environmental conditions, such as low illumination and shadow interference. Additionally, the inference latency per frame is recorded on the testing platform to isolate the computational overhead of the perception layer from the subsequent logic layers.

Hyperparameter Sensitivity Analysis: To rigorously justify the empirical thresholds within the proposed geometric and clustering layers, a systematic sensitivity analysis is conducted. The analysis evaluates two core hyperparameters: the outlier rejection multiplier λ for linear topology reconstruction and the longitudinal connectivity threshold δ for 1D longitudinal clustering. The optimal values are determined by analyzing their impact on the average angular fitting error and the topological parsing accuracy, respectively.

Validation Protocol for Geometric Perception Algorithms: A dedicated dataset encompassing various lighting conditions and dynamic disturbances is utilized to evaluate the effectiveness of the lightweight geometric perception algorithm. For straight-path scenarios, an evaluation subset of 244 typical samples is constructed to benchmark the proposed MAD-based robust line fitting against standard estimators, including ordinary least squares (OLS), PCA [18], Theil-Sen [19], and RANSAC [10]. The performance is quantitatively assessed based on the average angular fitting error and computational latency. For complex intersection topologies (L-shaped, T-shaped, and crosses), another evaluation subset comprising 134 frames is utilized to compare the proposed 1D connectivity clustering algorithm against standard clustering methods such as K-Means [13], Agglomerative clustering [14], Mean Shift [15], and DBSCAN [16]. This comparison focuses on evaluating the topological parsing accuracy and the corresponding processing overhead. Effective parsing strictly requires a correct branch count and a total angular deviation $\leq 10^{\circ }$. To rigorously isolate the algorithmic computational burden, the latency metrics reported in this phase specifically measure the execution time of the CPU-bound logic layer, explicitly excluding the inference overhead of the GPU-accelerated perception layer.

Frame-Level Accuracy Evaluation of Deviation Warnings: To evaluate the frame-level accuracy of the geometric deviation warning algorithm, a geometric evaluation dataset comprising 459 frames is established. Manually annotated tactile paving centerlines and topological branches serve as the ground truth. For straight-path linear fitting, a prediction is considered accurate if the fitted line falls within the actual tactile paving with an angular deviation of $|\Delta \theta |<5^{\circ }$. The evaluation of topology parsing for complex intersections follows the previously established metrics.

Robustness Validation Protocol for Dynamic Interactions: To assess the temporal robustness over continuous sequences, a dedicated dataset encompassing obstacle avoidance, stair negotiation, and street crossing is curated to evaluate the stability of FSM state transitions. This continuous sequence evaluation is purposefully designed as a targeted dynamic temporal stress test for system-level validation, with subsequent study phases planned to involve visually impaired participants (Section 6.3). The dataset comprises six manually curated continuous frame sequences extracted from videos collected by the research team, each covering a complete interaction event, totaling 1260 frames. Specifically, these encompass dynamic obstacle avoidance (2 sequences), stair negotiation (2 sequences), and street crossing (2 sequences). These sequences were deliberately selected to incorporate challenging state entry and exit conditions, alongside severe mid-process disturbances such as sudden tactile paving interruptions, transient visual occlusions, and multi-FSM preemption scenarios. An event-centric evaluation is conducted to examine the robustness of the FSM transition logic and state stabilization mechanisms under these dynamic environmental disturbances. This rigorously verifies the timeliness of state transitions, as well as the system’s temporal stability, logical coherence, and noise-reduction capabilities in preventing premature state rollbacks.

Computational Efficiency Evaluation: A continuous test sequence comprising 1134 frames under representative scenarios is curated to evaluate the real-time performance and quantify the empirical computational overhead. This sequence covers five core scenarios: straight tactile paving, complex intersections, obstacle avoidance, stair negotiation, and street crossing. The overall computational latency and resource utilization are quantitatively assessed under a uniform input resolution of 640×640. To characterize the computational distribution, the processing pipeline is decomposed into three logical modules: a global perception layer for object detection, an event-driven scenario-specific processing layer responsible for topology reconstruction and geometric computation in straight and intersection scenarios, and a warning logic layer that serves as the central decision module. This layer performs geometric deviation estimation and topological clustering, manages FSM state transitions with noise filtering and stabilization mechanisms, and coordinates priority arbitration and directive generation for multi-scenario warnings. Frame rates, per-module latency, peak GPU memory usage, and total system power draw are actively monitored to validate the feasibility of edge deployment.

## 5. Results

In accordance with the experimental procedures outlined in Section 4, this section presents the objective evaluation results of the proposed assistive system across complex real-world scenarios. The quantitative and qualitative findings are sequentially reported across six key dimensions: performance evaluation of the perception model, hyperparameter sensitivity analysis, validation of the geometric perception algorithm, frame-level accuracy evaluation of geometric deviation warnings, robustness validation of dynamic multi-scenario interactions, and computational efficiency evaluation.

### 5.1. Performance Evaluation of the Perception Model

The perception model achieves an mAP@0.5 of 72.1% on the validation set and yields an inference latency of 15.1 ms per frame on the testing platform. This measurement establishes a precise computational baseline, enabling the additional processing overhead introduced by the subsequent geometric logic layer to be distinctly isolated and quantified.

Quantitative metrics for simplified categories are presented in Table 1. The model demonstrates high recall rates on targets such as stairs and traffic lights, providing safety redundancy for high-risk scenarios. For core tactile tile targets, the detection results exhibit high precision but low recall. This is primarily attributed to the dense “instance-level” annotation strategy; under real-world conditions involving severe wear and occlusions, the model inevitably misses some individual small tiles. To capture dense geometric cues and alleviate spatial semantic sparsity, the inference stage is configured to lower the confidence threshold, which inherently introduces unstructured background noise. This observation underscores the necessity of the subsequent geometric reconstruction algorithm. Acting as a robust geometric filter, it effectively compensates for these local perception misses by filtering spatial outlier noise and establishing topological connections among discrete tactile tiles to extract a clean macroscopic skeleton.

**Figure 5:**
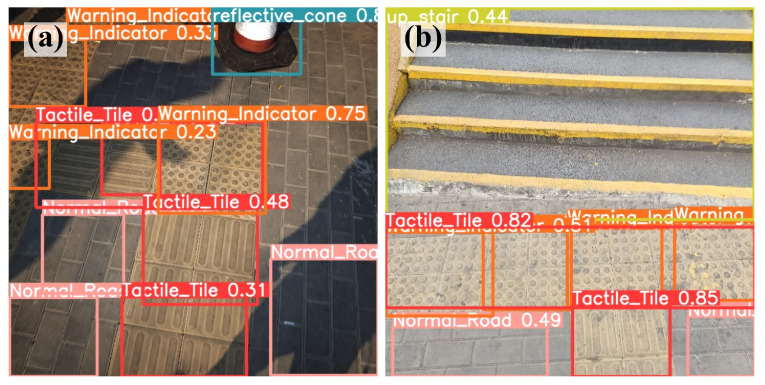
Qualitative detection results in real-world scenarios: (**a**) accurate detection of tactile tiles and a reflective cone under strong shadow interference; (**b**) localization of ascending stairs and the critical buffer zone.

Qualitative evaluations in complex real-world scenarios (Figure 5) further demonstrate the robustness of the front-end perception model. Under nighttime low-light conditions and strong shadow interference (Figure 5a), the model successfully identifies static obstacles such as reflective cones and produces well-localized bounding boxes for discrete tactile tiles. The model also accurately localizes the critical buffer zone leading to the ascending stairs (Figure 5b). These stably extracted semantic primitives directly serve as the data inputs for the subsequent geometric reconstruction and FSM state evaluation.

### 5.2. Hyperparameter Sensitivity Analysis

**Figure 6:**
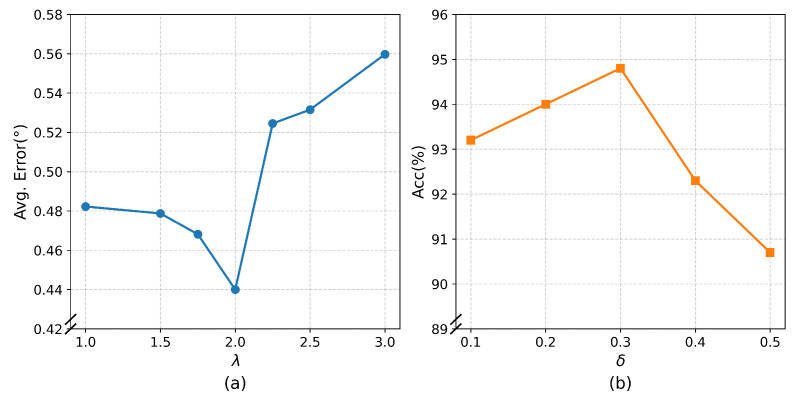
Hyperparameter sensitivity analysis curves: (**a**) impact of the linear topology outlier rejection multiplier λ on the average angular fitting error (deg); (**b**) impact of the longitudinal connectivity threshold δ on the topological parsing accuracy (%).

The corresponding geometric and topological accuracy validation curves are illustrated in Figure 6.

As shown in Figure 6a, the average angular error of the linear topology fitting varies with the multiplier λ. When λ increases from 1.0 to 1.75, the fitting error changes from $0.482^{\circ }$ to $0.468^{\circ }$. The minimum error of $0.440^{\circ }$ is attained at λ=2.0. For λ≥2.25, the error increases to $0.532^{\circ }$ and above due to the inclusion of background spatial noise. Conversely, for the directional branches with warning indicators (Section 3.2), a larger multiplier 2.5 is qualitatively selected to provide structural redundancy, as intersection areas present higher visual complexity than straight segments.

Figure 6b plots the topological parsing accuracy against the longitudinal connectivity threshold δ. The maximum accuracy of 94.8% is achieved at δ=0.3 m. Thresholds below this (δ≤0.2 m) lead to structural over-segmentation with accuracies between 93.2% and 94.0%, while larger thresholds (δ≥0.4 m) result in under-segmentation, reducing the accuracy below 90.7%. Additionally, a fusion threshold ϵ=0.2 m, which is smaller than δ=0.3 m, is utilized to prevent the merging of adjacent bifurcations. This configuration of parameters (λ=2.0, λ=2.5, δ=0.3 m, ϵ=0.2 m) is maintained constant for the subsequent evaluations.

### 5.3. Validation of the Geometric Perception Algorithm

#### 5.3.1. Comparison of Robust Line Fitting Algorithms

For straight tactile paving, detection point sets frequently suffer from outlier noise. Comparative tests on 244 typical samples (Table 2) demonstrate that standard estimators, including ordinary least squares, PCA [18], and the Theil-Sen estimator [19], lack adequate robustness. Although RANSAC [10] achieves the lowest angular error ($0.42^{\circ }$), it incurs a high computational latency of 1.30 ms. In contrast, the proposed MAD-based strategy achieves a comparable error ($0.44^{\circ }$) in merely 0.26 ms, delivering a five-fold speedup over RANSAC.

#### 5.3.2. Comparison of Clustering Algorithms for Intersection Topologies

Experiments (Table 3) show that K-Means [13] achieves high accuracy (94.0%) but incurs substantial latency (34.75 ms). Agglomerative clustering [14] is computationally efficient (0.55 ms) but yields a lower accuracy of 84.4%. Distance threshold-based methods, including Mean Shift [15] and DBSCAN [16], yield accuracies below 86%. In contrast, the proposed 1D connectivity clustering algorithm achieves the highest accuracy among the evaluated methods (94.8%) while reducing latency to 0.22 ms.

### 5.4. Frame-Level Accuracy Evaluation of Geometric Deviation Warnings

Quantitative results are presented in Table 4. The system achieves an accuracy of 96.3% in straight-path scenarios and 94.0% in intersection scenarios. These metrics indicate that the proposed geometric reconstruction algorithms can reliably extract path centerlines and resolve topological branches under real-world conditions.

### 5.5. Robustness Validation of Dynamic Multi-Scenario Interactions

Quantitative results are summarized in Table 5. For the obstacle avoidance FSM, the average triggering distance reaches 2.15 m. Furthermore, the total state reset latency is measured at 2.6 s (26 frames). Excluding the 2.0 s (20 frames) mandatory hysteresis buffer imposed by the state stabilization mechanism, the effective topology recapture latency is recorded at 0.6 s (6 frames). For stair negotiation scenarios, the system records an average trigger distance of 2.78 m. Notably, zero premature state rollbacks are observed across all test sequences. In street crossing scenarios, the system successfully captures 100% of the end-of-phase green light blinking events. Concurrently, a pedestrian-following activation rate of 94.9% is achieved during the crossing phase.

### 5.6. Computational Efficiency Evaluation

**Figure 7:**
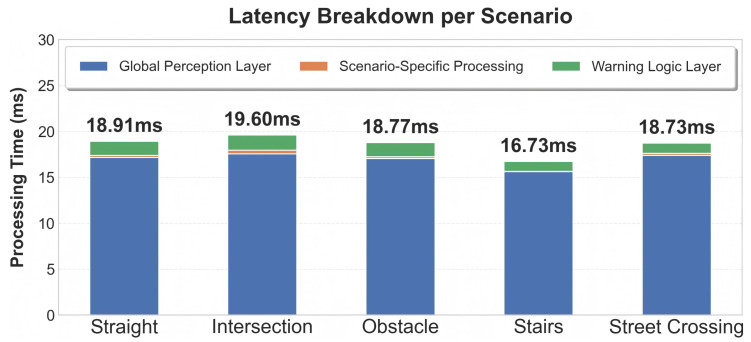
Per-frame latency by scenario.

The per-frame processing time for each scenario is detailed in Figure 7. On the testing platform, the system achieves an average frame rate of 53.24 FPS. A detailed latency breakdown reveals that the global perception layer maintains an inference time between 15 and 18 ms. Crucially, the core geometric and logic modules, which execute entirely on the CPU, introduce only 1.0 to 2.0 ms of additional overhead. Even under intersection scenarios involving complex topological branching, the peak per-frame latency is recorded at 19.60 ms. Furthermore, the peak GPU memory usage for multi-scenario interactions stabilizes at 307.36 MB. This extreme efficiency is further corroborated by empirical system monitoring: the entire pipeline, inclusive of background Windows operating system processes and concurrent applications, required only approximately 11% total CPU utilization (operating at ∼3.6 GHz, remaining well below the processor’s peak boost capacities). Additionally, while the baseline idle GPU power consumption of the testing platform was measured at 39 W, the total system-level GPU power draw during continuous inference stabilized at 50 W. This demonstrates that the dynamic power overhead directly attributable to the front-end perception model is a mere 11 W.

## 6. Discussion

The experimental results demonstrate that the proposed lightweight assistive system achieves an excellent trade-off between perceptual accuracy and computational efficiency, yielding a global warning accuracy of 95.21%. Compared to computationally intensive end-to-end architectures that require substantial resources for deep scene understanding, the proposed hierarchical framework effectively distills complex spatial information into concise geometric logic. By bridging the gap between discrete visual primitives and continuous temporal interactions, this system establishes a robust mathematical and logical foundation for both feasible directional prompts and state-adaptive warnings, delivering reliable mobility assistance and environmental awareness across varied real-world scenarios.

### 6.1. Effectiveness of Geometric and Temporal Reasoning

The comprehensive evaluation demonstrates that the proposed geometric reasoning algorithm achieves a favorable balance between high frame-level accuracy and computational efficiency in unstructured environments. By tailoring specific algorithmic strategies to the unique geometric features and topological types of tactile paving, the system ensures highly reliable spatial parsing. For straight tactile paving, standard robust estimators like RANSAC [10] introduce latency fluctuations due to their non-deterministic iterative nature. Conversely, the proposed MAD-based robust fitting algorithm guarantees bounded execution time owing to its O(N) linear complexity, acting as a highly efficient spatial filter against outlier noise caused by uneven illumination or tactile tile wear. For complex intersections, conventional distance-based clustering algorithms (e.g., Mean Shift [15] and DBSCAN [16]) are inherently sensitive to depth-dependent density variations in monocular point clouds. By leveraging a perspective-aware longitudinal distribution prior, the proposed customized 1D connectivity clustering algorithm reformulates the 2D problem into a 1D ordered process with O(NlogN) complexity, successfully resolving the semantic ambiguities that standard density-based methods typically encounter at multi-branch intersections. Crucially, for the sparse point sets (N≤20) typical in these regions, this stateless and non-iterative design fundamentally eliminates the heavy constant-factor overheads (e.g., spatial index initialization and distance matrix allocation) of traditional frameworks, achieving an ultra-low empirical latency of just 0.22 ms.

Beyond static geometric perception, continuous sequence evaluations highlight the system’s dynamic temporal robustness. The implemented FSMs effectively mitigate risks across safety-critical dynamic scenarios. In obstacle avoidance, the average triggering distance of 2.15 m significantly exceeds the minimum braking safety margin of 1.0 m, providing ample collision avoidance redundancy alongside an effective topology recapture latency of merely 0.6 s. For stair negotiation, which strictly demands temporal stability, the 2.78 m trigger distance offers a sufficient spatiotemporal margin for gait adjustment. The suppression window incorporated within the temporal hysteresis design completely prevents premature state rollbacks and suppresses false positives, ensuring a stable negotiation process. Finally, in complex street crossing scenarios, the system reliably captures 100% of green light blinking events. Crucially, the 94.9% pedestrian-following activation rate demonstrates the effectiveness of the proposed concurrent ground perception strategy. This behavior-level redundancy, enabled by dual-camera synergy, intelligently leverages crowd behavior priors to compensate for perceptual blind spots caused by transient traffic signal loss.

### 6.2. Computational Efficiency and Feasibility for Edge Deployment

A core objective of this study is to provide an architecture viable for real-time deployment on resource-constrained edge platforms. As noted in Section 4.1, while the system was evaluated on a high-performance workstation, this setup was strategically selected to establish a strict computational baseline and isolate the processing latency of the CPU-bound logic layer. Although there is an inherent disparity in raw computational power between the testing workstation and modern edge AI platforms (such as the NVIDIA Jetson Orin series), deploying deep learning perception models on these edge devices typically utilizes hardware-specific acceleration to guarantee real-time performance. For example, recent studies have demonstrated the effectiveness of utilizing NVIDIA’s TensorRT framework and INT8 quantization to optimize YOLO models for real-time inference on Jetson platforms [20], as well as employing specialized Neural Processing Units (NPUs) or ultra-low-power RISC-V processors for onboard detection [21]. Therefore, since the perception model’s inference can be efficiently offloaded to edge accelerators, the primary concern for architectural viability is the performance overhead of the non-accelerable logical components.

The detailed latency breakdown demonstrates that the scenario-specific geometric processing and FSM warning logic execute entirely on the CPU with minimal computational cost (averaging 0.22 ms for geometric computations and 1.38 ms for warning logic), thereby limiting the total logic overhead to approximately 1–2 ms. Compared to computationally intensive dual-branch semantic segmentation networks [5] or architectures that struggle to balance on-device deployment with complex perception [3], the proposed hierarchical architecture elegantly circumvents the computational bottlenecks typically encountered in dynamic multi-scenario assistance. For instance, recent VLM approaches [3] still exhibit inference latencies exceeding 29 s and peak memory footprints of around 760 MB on mobile hardware, even when employing 500 M-parameter models quantized to INT8. This limitation fundamentally stems from the high computational cost of autoregressive token generation. Given that the front-end perception model consumes only 15.1 ms on the testing baseline with a minimal dynamic GPU power overhead of 11 W, and the logic layer introduces negligible computational burden (operating at only 11% total system CPU utilization), the proposed system preserves sufficient computational headroom. Combined with a low memory footprint stabilizing at 307.36 MB, these metrics robustly verify the system’s theoretical and practical feasibility for edge deployment without requiring specialized logical acceleration. Ultimately, this performance effectively bridges the inherent gap between lightweight model design and deep semantic perception.

### 6.3. Limitations and Future Work

Despite high overall performance, a detailed analysis of failure cases reveals certain inherent limitations of monocular vision-based geometric reasoning. Approximately 3.7% of straight-path fitting failures occur when the tactile paving lies near the extreme margins of the visual field, resulting in spatially sparse observations. A minimal number of anchor boxes (N<3) leads to angular drift in the fitting algorithm due to insufficient sampling points. Furthermore, about 6.0% of intersection misjudgments occur at zigzag turning intersections, where curvature variation caused by turning and subsequent straightening allows a single physical branch to span multiple decision regions in the image space. Without global temporal reasoning based on connectivity, static clustering may incorrectly segment such structures into multiple independent paths.

Additionally, the robustness of the perception layer remains susceptible to severe environmental disturbances. The previously observed low recall for tactile tiles is primarily driven by physical surface wear, complex illumination, and visual occlusion. Extreme lighting conditions (such as intense backlighting or strong shadows), texture loss due to water accumulation, and heavy occlusion in dense crowds can degrade bounding box regression and disrupt the availability of geometric priors, occasionally preventing the system from performing stable topological reconstruction.

Furthermore, a current limitation of the temporal interaction evaluation is that the FSM transition logic is validated through a limited number of video sequences representing typical high-risk scenarios. While this targeted stress testing rigorously proves the logical coherence and state-stabilization capabilities of the system under severe dynamic disturbances, it lacks comprehensive statistical analysis based on large-scale field evaluations involving visually impaired participants. Additionally, while theoretical latency evaluations demonstrate the system’s extreme computational efficiency, it has not yet undergone real-time deployment and hardware validation on dedicated edge platforms.

To address these challenges while maintaining the objective of lightweight deployment, future research will focus on three main dimensions. First, the next stage of this research will focus on deploying the system on physical edge hardware and conducting extensive real-world evaluations with visually impaired participants to assess long-term temporal robustness and user interaction performance. Crucially, these evaluations will consider user diversity, as visually impaired individuals vary significantly in walking speed, white-cane swinging amplitudes, and cognitive preferences. To accommodate this heterogeneity, future iterations will transition from the current “one-size-fits-all” weak-intervention strategy toward a more personalized assistive system, incorporating customizable parameters such as adjustable auditory interaction frequencies, tailored tactile-paving deviation sensitivities, and adaptive warning distance thresholds. Second, advanced model compression strategies, such as knowledge distillation and structured pruning, will be implemented to further enhance energy efficiency and tailor the perception backbone for specific edge NPUs. Third, to compensate for transient visual degradation, multi-modal sensor fusion incorporating Inertial Measurement Units (IMUs) will be explored. By leveraging kinematic priors to maintain spatiotemporal consistency, the system can provide more reliable and continuous directional prompts across diverse and adverse real-world scenarios.

## 7. Conclusions

This paper proposes a lightweight real-time assistive system that bridges the gap between discrete visual perception and high-level cognitive reasoning through a hierarchical architecture. Instead of relying on heavy end-to-end visual models for deep scene understanding, the system utilizes a lightweight model solely to extract discrete semantic primitives, upon which an independent layer integrating geometric topology reconstruction and state-adaptive warning logic is constructed. This architecture successfully resolves the inherent conflict between requiring deep logical reasoning and adhering to edge computational constraints. Furthermore, it achieves a precise transition from low-level discrete visual primitives to high-level macroscopic directional semantics, establishing a unified synergistic framework encompassing topology reconstruction, scene-adaptive warning, and multi-task temporal interaction. Comprehensive evaluations indicate that the system achieves a warning accuracy of 95.21% under complex dynamic conditions. Substantiated by both theoretical algorithmic complexity and actual empirical evaluations, the minimal computational overhead of the core logic layer ensures high inference efficiency, thereby validating the feasibility of deploying the system on resource-constrained edge platforms. It provides solid performance support for delivering safe directional prompts to visually impaired individuals in their immediate complex surroundings.

## Figures and Tables

**Table 1:** Quantitative results of the perception model.

Group	Class Name	P	R	mAP50
Tactile	Tactile Tile	0.76	0.25	0.473
Paving	Warning Indicator	0.55	0.38	0.410
	Person (Obstacle)	0.69	0.56	0.610
Guidance	Car (Obstacle)	0.62	0.63	0.628
Context	Traffic Lights *	0.87	0.94	0.937
	Stairs ^†^	0.95	0.98	0.990
**Overall**	**All 24 Classes**	**0.76**	**0.67**	**0.721**

* Traffic lights include red, green, and pedestrian lights. ^†^ Stairs include both up_stair and down_stair categories.

**Table 2:** Comparison of line fitting algorithms on real-world scenarios.

Algorithm	Avg. Error (°)	Time (ms)	Complexity
OLS (Baseline)	0.59	**0.13**	O(N)
PCA [18]	0.93	0.23	O(N)
THEILSEN [19]	1.03	0.35	$O(N^{2})$
RANSAC [10]	**0.42**	1.30	O(K·N)
**Ours (MAD-based)** [9]	0.44	0.26	O(N)

**Table 3:** Performance comparison of intersection topology clustering.

Method	Config	Acc. (%)	Time (ms)	Complexity
K-Means [13]	K=3	94.0	34.75	O(I·K·N)
Agglomerative [14]	n=2	84.4	0.55	$O(N^{2}\log N)$
Mean Shift [15]	bw=0.2	85.1	89.20	$O(I\cdot N^{2})$
DBSCAN [16]	ϵ=0.15	81.3	12.07	O(NlogN)
**Ours**	δ=0.3	**94.8**	**0.22**	O(NlogN)

**Table 4:** Frame-level evaluation metrics for path deviation warnings.

Scenario	Total	Correct	Acc. (%)
Straight Path	244	235	96.3
Intersection	215	202	94.0

**Table 5:** Key performance metrics for multi-scenario dynamic interactions.

Scenario	Performance Metric	Value
Obstacle Avoidance	Trigger Distance	2.15 m
	Recovery Latency	<2.6 s
Stair Negotiation	Trigger Distance	2.78 m
	Premature Rollbacks	0
Street Crossing	Green Blinking Capture	100%
	Pedestrian Following Rate	94.9%

## Data Availability

The data presented in this study are available on request from the corresponding author. The data are not publicly available due to the large volume of individual files and privacy considerations associated with real-world road scenarios.

## Abbreviations

The following abbreviations are used in this manuscript:

ETA	Electronic Travel Aids
FSM	Finite State Machine
MAD	Median Absolute Deviation
MST	Minimum Spanning Tree
ROI	Region of Interest
DFS	Depth-First Search
TGSI	Tactile Ground Surface Indicators
